# Metachronous Bilateral Adrenal Metastasis From Renal Cell Carcinoma Following Robot-Assisted Partial Nephrectomy: A Case Report

**DOI:** 10.7759/cureus.85143

**Published:** 2025-05-31

**Authors:** Oluchi Idenyi, Vishwanath Anil, Rao Moravineni, Sunil Shahi, Nageshwar Kothur, Winston Ugbajah, Daniel Assaf, Onyekachi Anya, Getnet Tioum

**Affiliations:** 1 Internal Medicine, Wellstar Spalding Regional Hospital, Griffin, USA; 2 Medical Oncology and Hematology, Wellstar Spalding Regional Hospital, Griffin, USA; 3 Pathology, Wellstar Spalding Regional Hospital, Griffin, USA; 4 Internal Medicine, Legacy Salmon Creek Medical Center, Vancouver, USA

**Keywords:** bilateral adrenal metastasis, metachronous, partial nephrectomy, rcc, robot assisted

## Abstract

Renal cell carcinoma (RCC) can recur or metastasize years after nephrectomy. While it is uncommon for RCC to metastasize to the adrenal glands, bilateral involvement is very rare and often indicates a more aggressive form of the disease with a poorer prognosis. Therefore, long-term surveillance following nephrectomy is crucial and may improve patients’ outcomes. Here, we present a case of a 63-year-old Caucasian male with a past medical history of hypertension, who underwent a robot-assisted right partial nephrectomy and was subsequently lost to follow-up. The pathology report revealed an unclassified renal cell carcinoma, stage 1 (PT1aN0M0). Post-operative imaging two months later showed no evidence of recurrence or metastasis. Despite initial successful management of the primary tumor, three years post-nephrectomy, the patient developed metastatic bilateral adrenal masses, distant metastasis, and rapid disease progression. We report this case to highlight the late metastatic potential of RCC, the rarity of bilateral adrenal involvement, and its associated poor prognosis, while underscoring the importance of post-surgical surveillance following resection.

## Introduction

Renal cell carcinoma (RCC) accounts for approximately 85% of all kidney cancer cases in adults and arises from the renal cortex due to abnormal proliferation of the tubular epithelium [1]. Common metastatic sites include the lungs (45.2%), bone (29.5%), lymph nodes (21.8%), and liver (20.3%) [2]. Despite the anatomical proximity of the adrenal glands to the kidneys, metastasis to these glands is relatively rare. A retrospective analysis of 610 patients with renal cell carcinoma (RCC) found that the incidence of ipsilateral adrenal metastasis was 3.4%, while contralateral adrenal metastasis occurred in 1.1% of cases. Among the contralateral cases, metastasis occurred simultaneously with the primary tumor (synchronously) in three patients, while it developed later (metachronously) in four others. Notably, three of the four metachronous cases showed bilateral adrenal involvement, underscoring the rarity of this condition, with an approximate incidence of 0.5%, and its association with late-stage disease progression [3]. Another retrospective study involving 1,635 patients who underwent radical nephrectomy found adrenal metastases in 90 patients (5.5%) with a false-negative rate of 20% on pre-operative imaging, highlighting diagnostic challenges [4]. Bilateral adrenal metastases are extremely rare, with only a few documented cases in the literature.

Current European Association of Urology (EAU) guidelines suggest that ipsilateral adrenalectomy should be considered in patients with tumors >4 cm or with advanced-stage disease (T3 or higher) because of a higher risk of adrenal involvement [5]. Yet, due to their rarity, there is no agreement on how to treat bilateral adrenal metastases. In isolated adrenal metastasis, some research has demonstrated that adrenalectomy can be of survival benefit when total resection is possible [6]. However, bilateral adrenalectomy carries a high risk of complications, including permanent adrenal insufficiency that necessitates long-term corticosteroid replacement [7]. We present a rare case of metachronous bilateral adrenal metastases in a patient with clear cell RCC following partial nephrectomy. Such presentations are scarcely reported, especially after nephron-sparing surgery. Clear cell histology is known for its aggressive behavior, tendency for distant metastases, and poor prognosis [8]. This case underscores the importance of vigilant follow-up, early imaging, and timely intervention in enhancing patient outcomes.

## Case presentation

A 63-year-old Caucasian male with a history of hypertension presented to the emergency department (ED) with complaints of severe nausea and non-bilious vomiting, experiencing more than five episodes within a 24-hour period. On presentation, the patient appears dehydrated, evidenced by dry mucous membranes and poor skin turgor. Laboratory examination was significant for acute kidney injury (AKI) with elevated levels of serum creatinine. Abdominal computed tomography (CT) showed an incidental finding of a right lower pole renal mass. Further evaluation with abdominal magnetic resonance imaging (MRI) revealed a lobulated, heterogeneous signal-intensity mass. The patient was stabilized with intravenous fluids and antiemetics. The patient's AKI resolved by the second day of admission. He was scheduled for an elective surgical procedure. The patient successfully underwent a robot-assisted right partial nephrectomy. The pathology report revealed unclassified renal cell carcinoma with no tumor invasion, classified as stage PT1aN0M0 (stage 1). Post-operatively, he was lost to follow-up.

Three years later, the patient presented to the emergency department (ED) with a sudden onset of nausea and non-bilious vomiting. Further history revealed loss of appetite, fatigue, and an unintentional weight loss of 40 pounds over two months. His vital signs were normal. A physical examination revealed a cachectic male who was not in acute respiratory distress.

**Figure 1:**
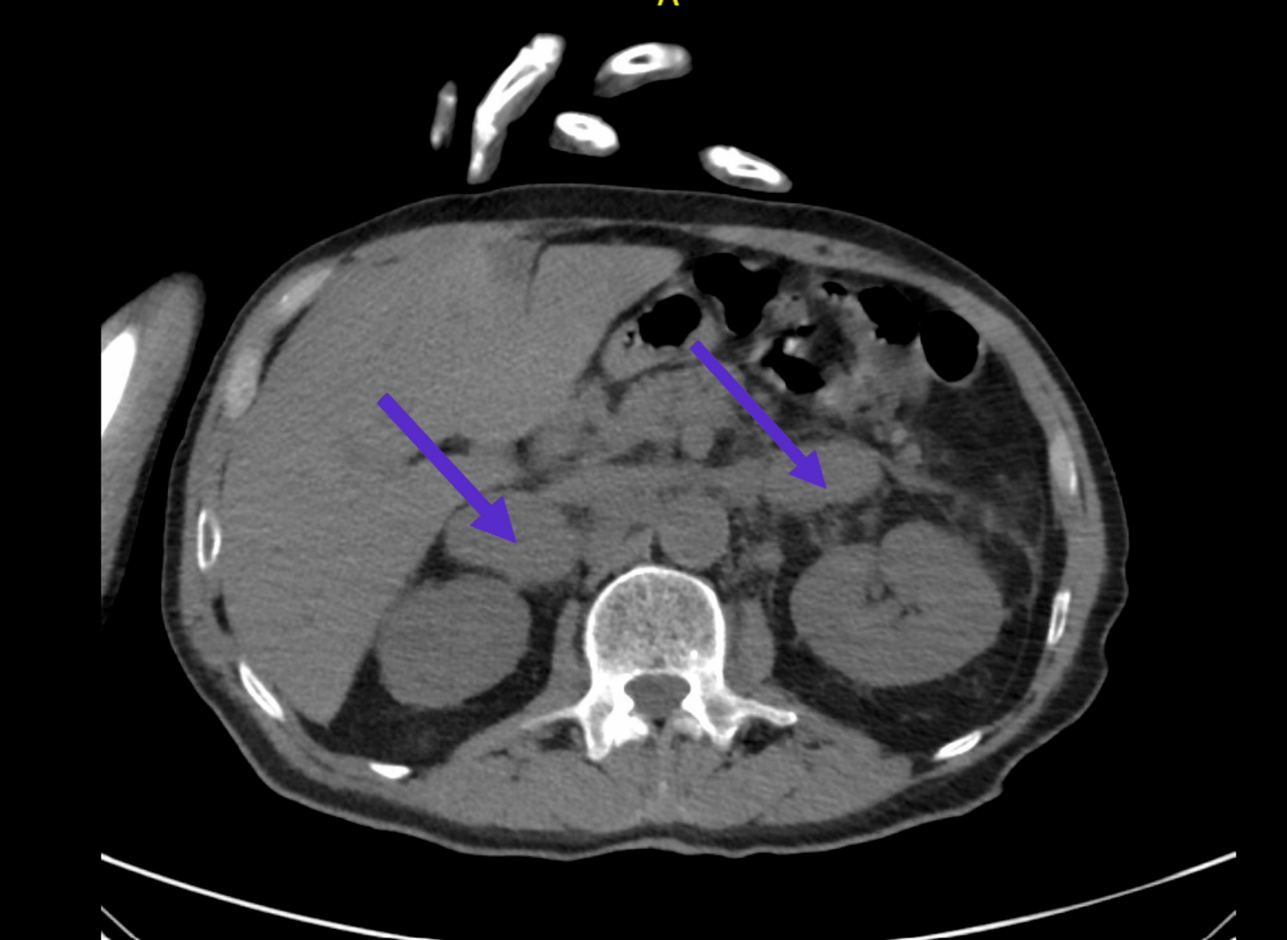
Bilateral adrenal masses (arrows) on non-contrast computed tomography (CT) abdomen and pelvis measuring 5.6 x 3.1 cm (right) and 3.3 x 3.2 cm (left)

On day one, laboratory tests revealed the following significant findings: low hemoglobin, an elevated white blood cell count (WBC), a high platelet count, and an elevated cortisol level (Table 1). These laboratory values remained unchanged until his discharge on day six. A non-contrast abdominal CT scan revealed bilateral adrenal masses (Figure 1). Hormonal evaluation indicated that the adrenal masses were nonfunctional.

**Table 1:** Initial blood tests obtained three years post-nephrectomy. The values remained insignificantly unchanged during the hospital stay.

Blood test	Result	Reference range	Notes
Hemoglobin	8.7 g/dL	13.5-17.5 g/dL	Significantly low, indicative of anemia
White blood count (WBC)	18.84 x 10^9^/L	3.50-10.50 x 10^9^/L	Elevated, indicating possible infection or inflammation
Platelet count	529 x 10^9^/L	150-450 x 10^9^/L	Elevated, may indicate an ongoing inflammatory response
Cortisol, 8 AM	19.4 µg/dL	6-18.4 µg/dL	Adrenal insufficiency ruled out

**Figure 2:**
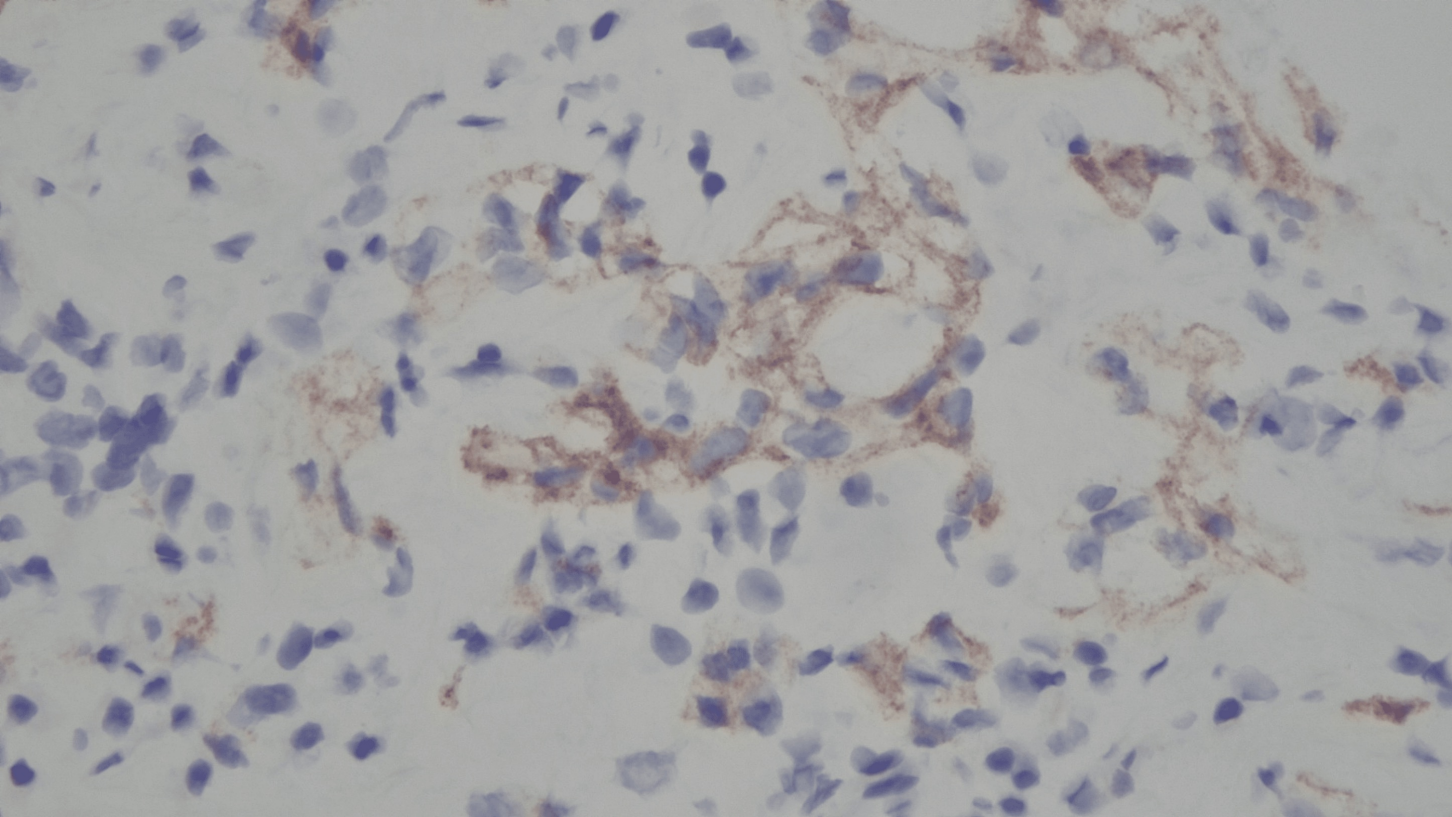
Metastatic clear cell renal cell carcinoma (CCRCC) showing presence of cells with clear cytoplasm (CAIX positive).

**Figure 3:**
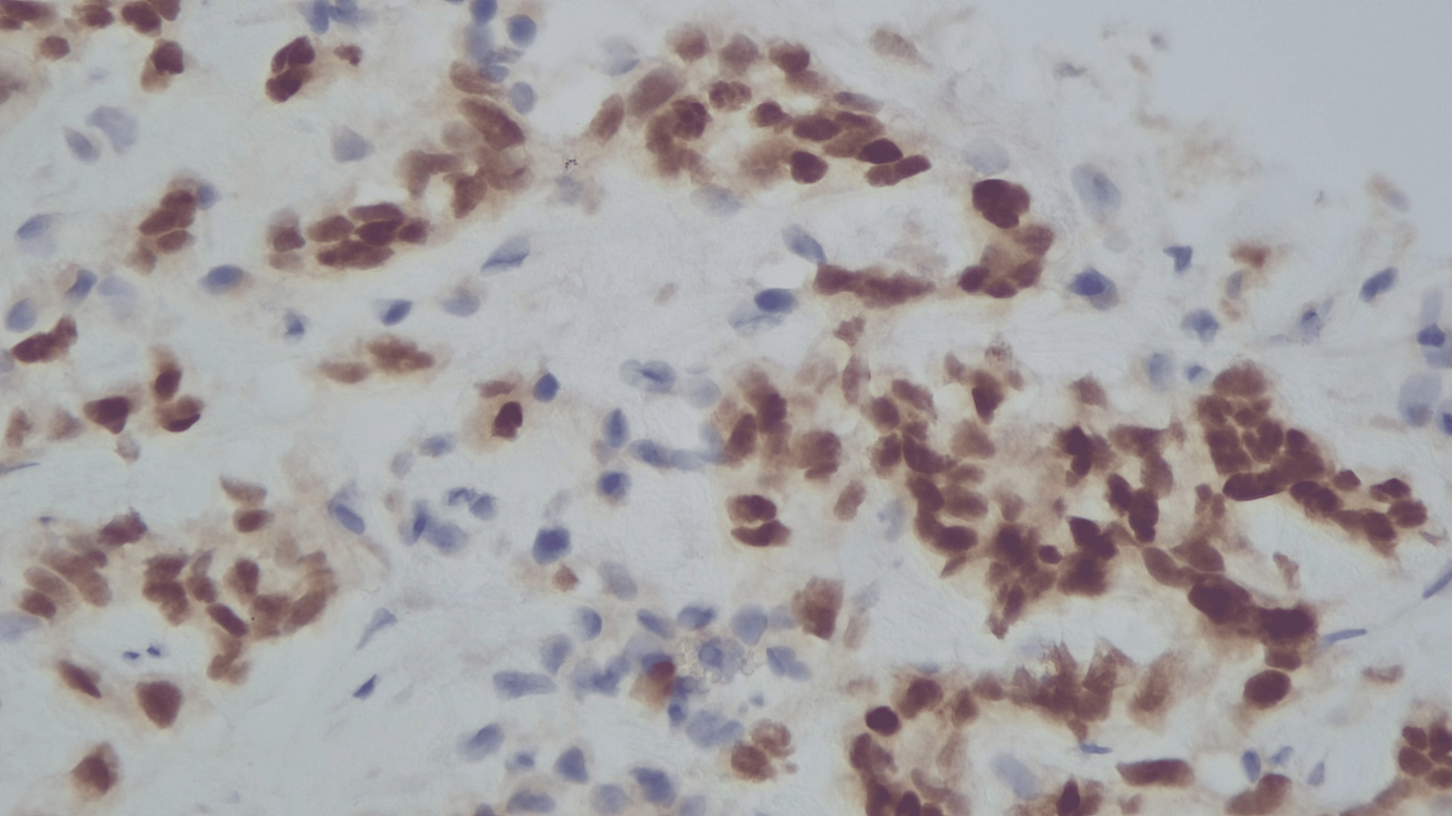
Immunohistochemical (IHC) examination showing metastatic CCRCC positive for PAX8 (nucleus). CCRCC: clear cell renal cell carcinoma

**Figure 4:**
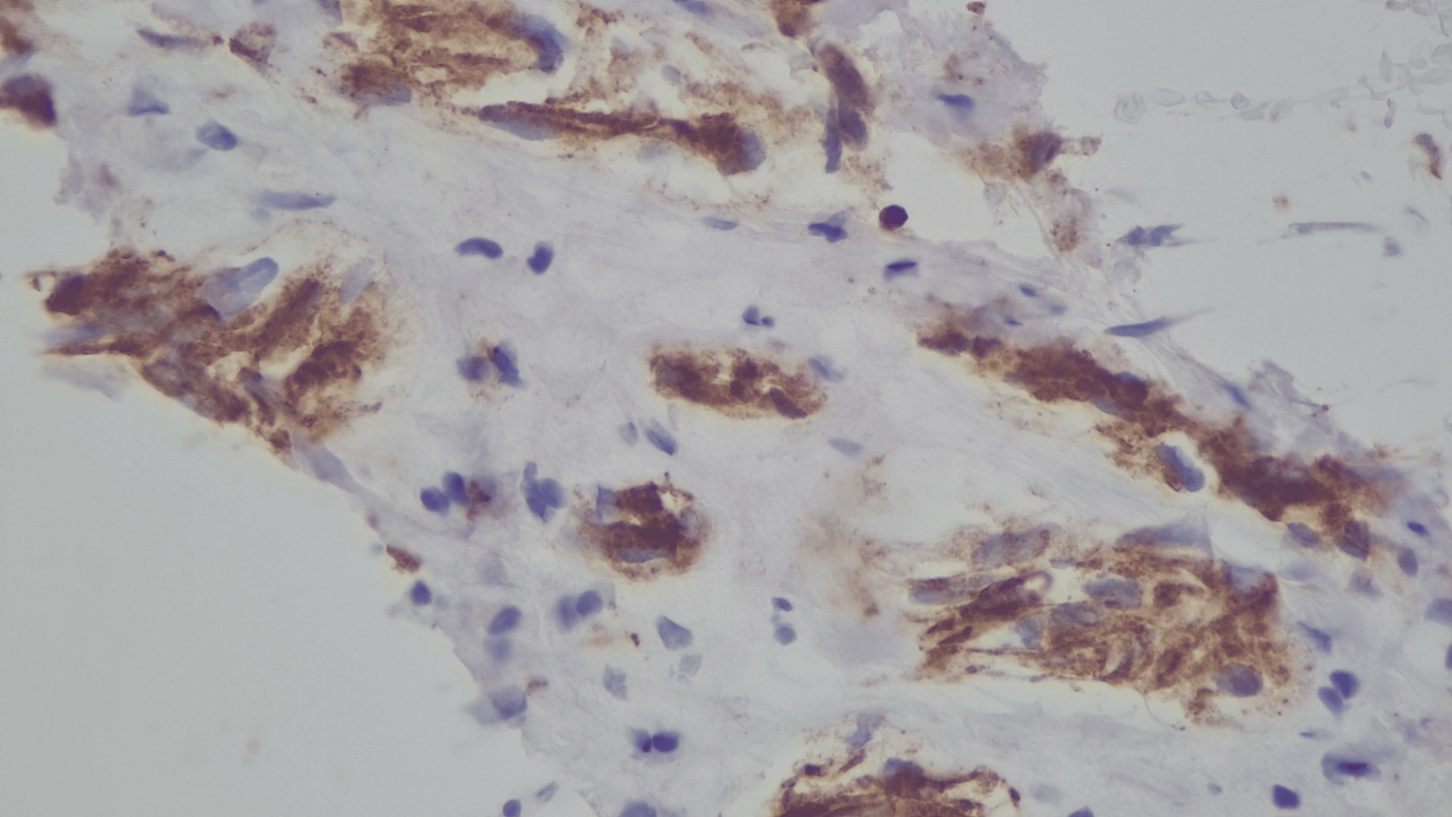
Immunohistochemical (IHC) staining positive for EMA (membrane). Findings suggestive of epithelial origin.

To further delineate the mass and evaluate other metastatic sites, a contrast-enhanced CT abdomen and pelvis was performed, revealing spine sclerotic foci, the largest at the T10 vertebral body, and a hypodense lesion of the liver and left kidney. Biopsy of the right adrenal mass revealed a metastatic clear cell renal cell carcinoma (CCRCC) grade 1 with immunohistochemical findings positive for CA 1X (cytoplasmic), PAX8 (nucleus), and EMA (membrane) (Figures 2-4).

The tumor cells were negative for CK7, CK20, CK68, ALK1, and SMA. No sarcomatoid features were noted. The patient was medically stable at discharge. An outpatient follow-up with oncology was scheduled.

**Figure 5:**
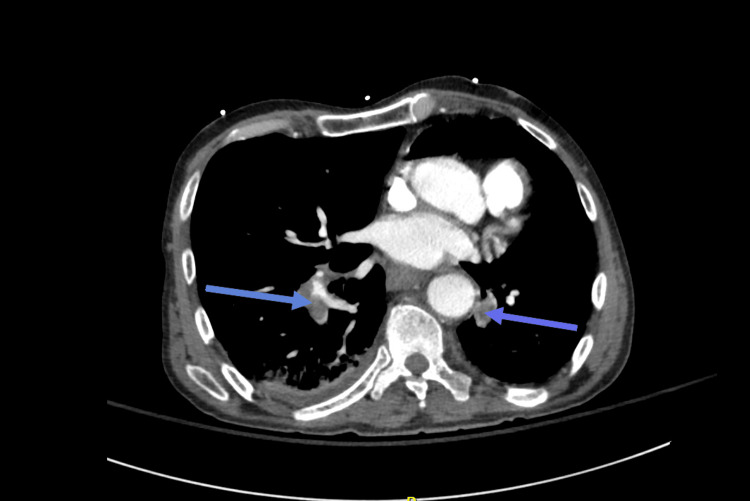
Multiple bilateral pulmonary embolism (arrows).

On post-discharge day 10, the patient re-presented to the ED with a sudden onset of nausea, abdominal pain, and a non-productive cough. Upon arrival, his blood pressure and heart rate were within normal limits, his respiratory rate was slightly elevated (22 cycles per minute), and his oxygen saturation was low on room air (86%), requiring oxygen supplementation via a nasal cannula. The physical examination showed an ill-looking male in mild respiratory distress. The laboratory studies were unchanged from his previous admission. A contrast CT angiography of the chest (CTA) revealed multiple bilateral pulmonary emboli (Figure 5).

**Figure 6:**
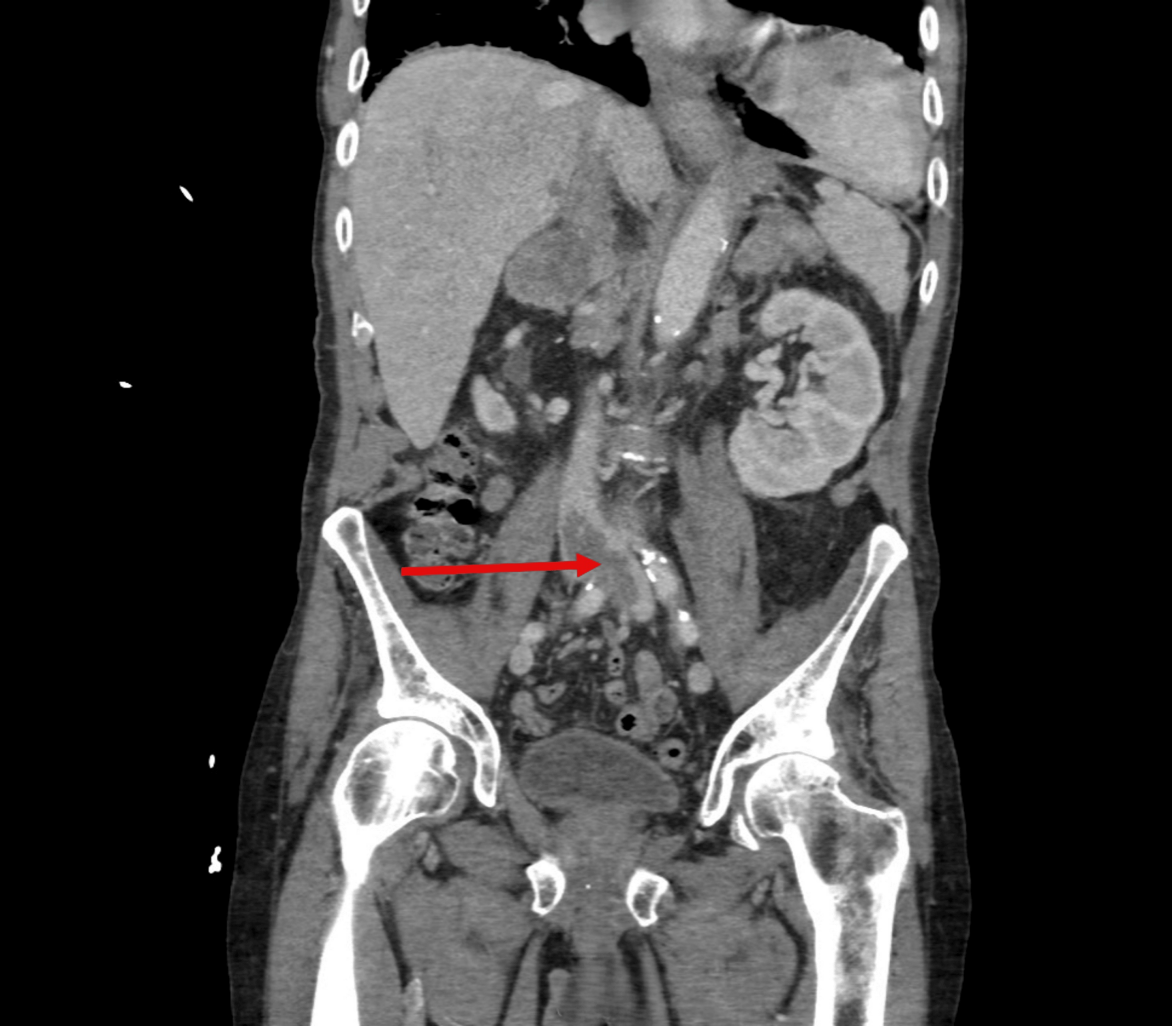
Coronal contrast CT abdomen showing a large IVC thrombus (arrow). IVC: inferior vena cava

**Figure 7:**
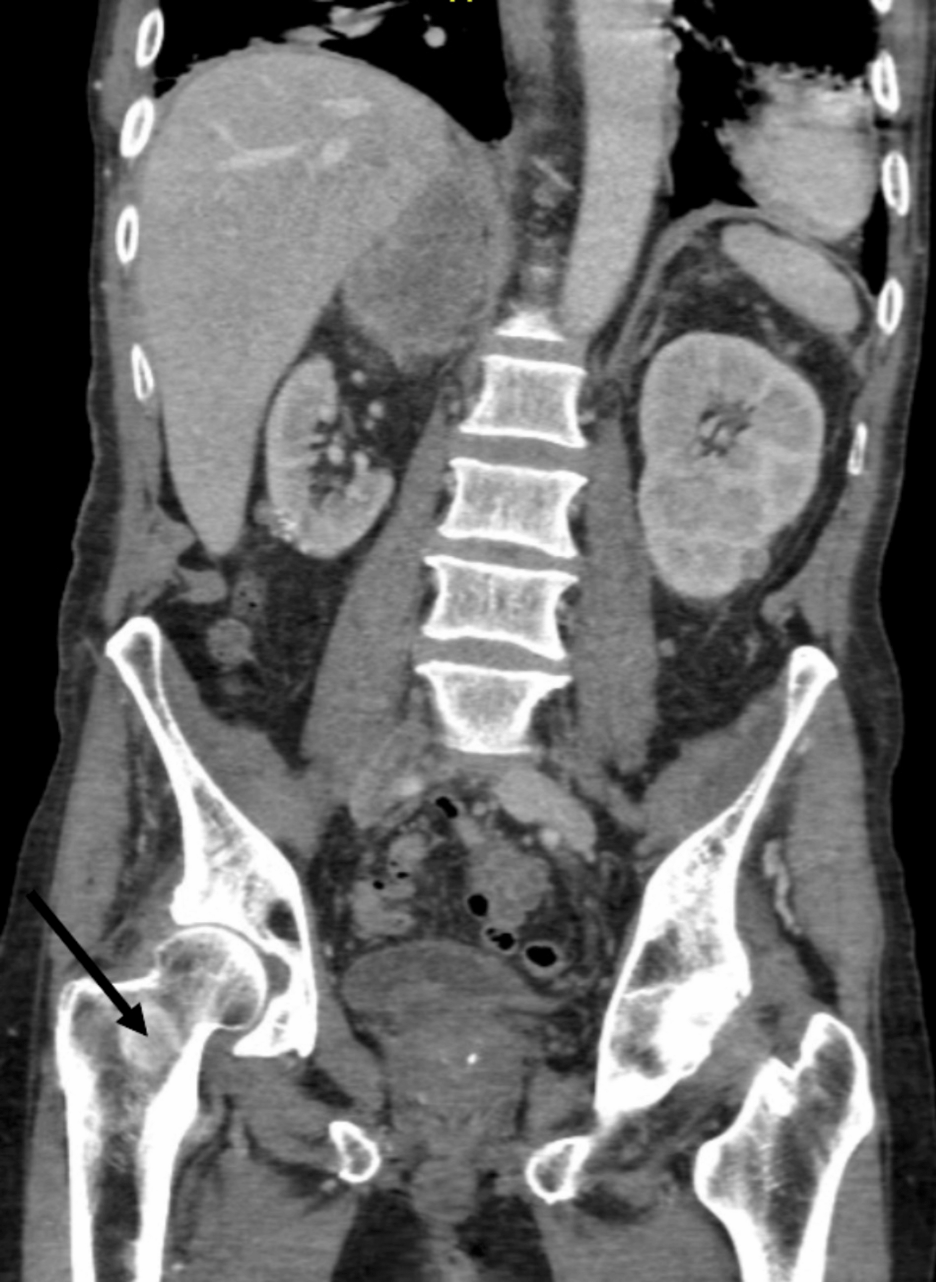
Sclerotic foci on the right proximal femur (arrow).

Further contrast-enhanced abdominal imaging examination revealed a large thrombus at the common iliac vein bifurcation of the distal inferior vena cava (Figure 6) and metastatic disease in the thoracic spine and right proximal femur (Figure 7).

The echocardiogram ruled out a patent foramen ovale (PFO) or right ventricular strain. The patient was started on antithrombotics with an intravenous heparin drip. Upon discharge, he required 2 L of home oxygen and was transitioned to oral apixaban. A follow-up appointment with outpatient oncology was scheduled. Table 2 represents the summary of the imaging results.

**Table 2:** Imaging report summary.

Imaging study	Findings	Notes
Non-contrast abdominal CT	Bilateral adrenal masses (5.6 x 3.1 cm on the right, 3.3 x 3.2 cm on the left)	Evidence of bilateral adrenal metastases
Contrast-enhanced abdominal CT	Spine sclerotic foci (T10 vertebral body), hypodense lesion in liver and left kidney	Indicating metastatic spread to multiple sites
Contrast-enhanced CT chest	Bilateral pulmonary emboli	Suggests significant thromboembolic events
Contrast-enhanced abdominal CT	Large thrombus at common iliac vein bifurcation of the distal inferior vena cava	Potential source of emboli leading to pulmonary embolism
CT abdominal imaging	Metastatic disease in spine and right proximal femur	Indicates widespread metastasis beyond adrenal glands

## Discussion

Adrenal metastasis from RCC can occur at the same time as the primary tumor (synchronous) or later (metachronous) after surgical resection of the primary tumor [9]. The exact mechanisms behind this process remain under investigation. Several studies have proposed potential pathways and contributing factors, including hematogenous spread, direct tumor invasion, and tumor dormancy, along with their associated characteristics. Occasionally, a patient who has been treated with partial nephrectomy for renal cell carcinoma may relapse near the surgical scar and progress to metachronous bilateral adrenal metastases, particularly in the absence of close follow-up [10]. In a study analyzing autopsy records of 1,828 patients with renal adenocarcinoma (424 of whom underwent nephrectomy and 1,404 who did not), there was no clear evidence that nephrectomy or adjuvant therapy improved outcomes. Instead, it concluded that the pattern of metastasis and disease progression in renal adenocarcinoma is driven more by the tumor's inherent biological characteristics than by surgical treatment [11].

Studies have shown that only about 1% of patients with RCC present with solitary adrenal metastasis without other metastatic sites at the time of diagnosis [12]. About 96% of cases in which adrenal metastases were found had advanced and disseminated disease [13,14]. Our patient experienced rapid disease progression and metastasis despite undergoing partial nephrectomy, highlighting the poor prognosis associated with bilateral adrenal involvement. While studies have indicated improved survival with early diagnosis and surgical intervention, these studies are very limited [15,16].

Most RCC recurrences occur within the first five years following surgery, suggesting intensive surveillance within this period [17]. Regular follow-up with clinical evaluation and imaging is recommended to detect and identify any metastatic or local recurrence features. Some studies have reported recurrences occurring five or more years after treatment. Our case underscores the importance of extended follow-up and an individualized approach to RCC surveillance after nephrectomy, in alignment with the most current surveillance guidelines [18,19].

A bilateral adrenal metastasis must be treated in a multidisciplinary fashion that involves the treatment of the primary tumor, the metastatic locations, and any related complications, such as thromboembolism. The fact that there are pulmonary emboli, IVC thrombi, bone, and spine involvement further demonstrates the severity and complexity of this patient's condition. Due to the patient's high tumor burden and overall performance status, along with the assessment that the patient was a poor surgical candidate, options for systemic therapy were discussed and offered, including but not limited to the risks and benefits. However, the patient declined further systemic therapy and opted for palliative care instead.

Metachronous bilateral adrenalectomy has proven to be an effective and practicable procedure in patients with no local or metastatic recurrence. However, this therapeutic intervention requires extensive experience and larger studies to prove this method [19,20]. We describe a rare case of metachronous bilateral adrenal metastases that developed from an initially unclassified, early-stage renal cell carcinoma. The observed histological transition to a clear cell subtype in the metastatic adrenal lesions suggests the potential for tumor heterogeneity or clonal evolution of the primary tumor, or it may reflect limitations in sampling. The absence of follow-up imaging delayed the detection of recurrence and limited our understanding of the timing and progression of metastatic spread. This underscores the importance of long-term surveillance post-nephrectomy in all patients with RCC, regardless of initial staging, to facilitate early detection and management of late metastatic recurrences.

## Conclusions

This case underscores the rare occurrence of bilateral adrenal metastasis from renal cell carcinoma following partial nephrectomy. Such cases are often associated with aggressive disease and poor prognosis. Despite an initial diagnosis at an early stage and a partial nephrectomy, our patient developed rapid disease progression years later, underscoring the tumor's biological heterogeneity and evolution. The lack of routine follow-up imaging delayed the early detection of recurrence or metastasis, emphasizing the need for long-term post-surgical surveillance, even in low-stage RCC. Follow-up of abnormal laboratory findings and routine imaging modalities, such as abdominal CT or MRI, at regular intervals, especially in the first few years post-surgery, is essential to detect recurrence or metastasis and may improve patient outcomes.
